# Application of Digital Techniques for the Observation and Fabrication of Removable Partial Dentures

**DOI:** 10.1155/crid/6565870

**Published:** 2026-08-05

**Authors:** Su Wu, Ting Pan, Zhaohan Du, Xinman Song, Shaohai Wang

**Affiliations:** ^1^ Department of Stomatology, Shanghai East Hospital, Tongji University School of Medicine, Shanghai, China, tongji.edu.cn

**Keywords:** chairside design, digital impression, digitized process, intraoral three-dimensional observation, RPD framework

## Abstract

This study explores digital fabrication of removable partial dentures (RPD) via direct intraoral model observation. At initial diagnosis, the three‐dimensional design scheme is obtained directly by visualizing the digital model using an intraoral scanner and a three‐dimensional management platform software. This framework supports tooth preparation, data acquisition, digital chair‐side observations, and RPD design and fabrication.

## 1. Introduction

With increasing aging population, the demand for dentition defect repair has increased [1]. To restore missing teeth in patients with dentition defects, clinicians widely use removable partial dentures (RPDs) because of their wide range of application, simple fabrication, and relatively low cost [2].

For traditional RPD fabrication process, patients require multiple appointments to prepare teeth, take impressions, record jaw positions, try in the framework, and make the adjustments and repairs necessary to improve the accuracy and suitability of the RPD. In clinical practice, doctors often roughly observe the intraoral situation after direct tooth mold preparation, which simplifies the treatment process to a certain extent; however, obtaining the ideal seating position is often difficult, causing errors in the fabricated dentures that necessitate time‐consuming adjustments and repairs.

The development of digital technology has led to gradual improvements in digital RPD manufacturing technology. However, the important step of model observation has not been digitally quantified; thus, to obtain accurate tooth undercutting data, it is necessary to prepare impressions and perfuse them into tooth plaster models and then fix the tooth plaster models on the observation platform for analysis and survey‐line marking. This step is not only tedious and time‐consuming but also difficult to change. Notably, digital impressions have good accuracy and can meet the needs of clinical application, resulting in their widespread use in the fabrication of fixed dentures and implant dentures [3–7]. Digital impressions may not replicate the mucosal shape under pressure and are therefore considered inappropriate for RPD preparation by some scholars. Digital impressions offer distinct advantages, including greater comfort, simplified operation, simultaneous occlusal relationship acquisition, long‐term data preservation, and the ability to repeatedly use the acquired data; therefore, an increasing number of scholars have begun to explore the feasibility of using digital impressions for RPD fabrication. Using digital impressions and direct model observations in a data management platform not only allows accurate observation data to be obtained but also enables model‐free RPD design and fabrication.

Several studies have shown that digital impressions can replace anatomical impressions, with good accuracy and reproducibility in Kennedy Class III and IV edentulous patients. Mansour et al. used digital impression technology and three‐dimensional printing technology to make Kennedy Class III tooth‐supporting RPDs [8]. The results revealed that patients were very satisfied with the restorative effect of the dentures, suggesting that the use of digital impressions to produce Kennedy Class III RPDs may improve RPD applicability without resulting in pressure on soft tissue. The use of digital impressions has also been reported in some studies of terminal‐free edentulism cases. Hu et al. reported that a RPD fabricated on the basis of oral scans of an initial impression in Kennedy Class I edentulous patient yielded satisfactory clinical restorative results [9]. The results of an experimental study by Zhong et al. revealed that the clinical suitability of RPDs fabricated via intraoral digital scanners could meet clinical application requirements [10]. Both Su and Huang reported the feasibility of digital impressions in Kennedy Class I and II patients [11, 12].

In this study, we analyzed the soft and hard tissues of the oral cavities of patients through physician‐side three‐dimensional management platform software (Vision 3D Big Data Intelligent Management Platform for Stomatology, 3.0.7). Afterwards, we completed the RPD design scheme and used it for intraoral tooth preparation. This method not only improves patient comfort but also enables the visualization and quantization of the patient′s intraoral condition. Therefore, the design scheme is more scientific and reasonable, eliminates model inversion and observation steps, and optimizes the overall process. In the software, we can directly analyze and observe the digital impression data, complete the filling undercut, draw wires, and preform the RPD according to the design scheme. The design plan used to facilitate tooth preparation after intraoral scanning can also be used to determine whether tooth preparation is reasonable, whether the space reserved for support is sufficient, and whether timely adjustment is needed. Directly recording the occlusal relationship in the patient’s mouth via intraoral scanning can not only eliminate the need for measurement of occlusion steps during traditional treatment, thereby reducing the number of patient visits, but also reduce the number of repairs and the chance of possible errors. Moreover, the software can connect with the technician side platform and directly transmit the collected data to the technician side for RPD production.

This study innovatively combined digital impressions, RPD design, and model observations to achieve an accurate design at the chairside, simplify the denture fabrication process, and facilitate communication between doctors and technicians. Moreover, the software database can provide a reference for junior doctors managing complex cases, effectively integrating clinical workflows with enhanced learning.

## 2. Case Presentation

**Figure 1:**
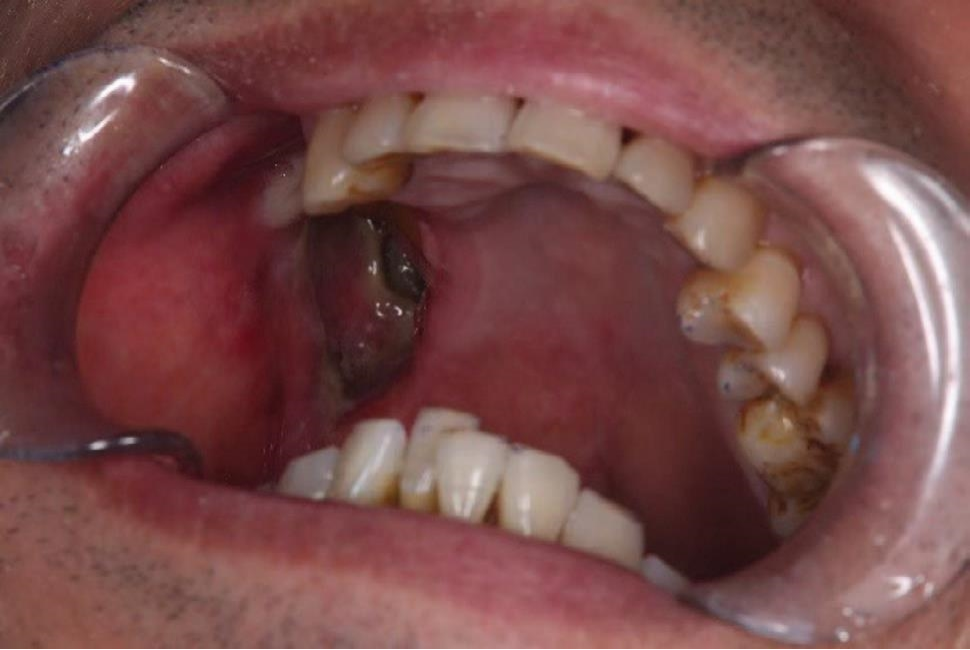
Intraoral soft and hard tissue defects in the patient.

A 67‐year‐old male patient who presented with ameloblastoma of the right maxillary sinus underwent “mass resection + partial resection of the right maxilla + opening of the right ethmoid and frontal sinuses” and underwent oral histomorphology and functional restoration because of right upper posterior tooth loss and partial absence of the maxilla (Figure 1). With the informed consent of the patient, a maxillary RPD was designed and fabricated according to traditional and digital processes.

### 2.1. Materials and Methods

#### 2.1.1. Personnel Qualifications

The RPD framework was designed by a physician with more than 5 years of experience and extensive training in the use of intraoral scanners and three‐dimensional management platform software. Three prosthodontists with 3, 5, and 8 years of experience managed the trial dentures, which were made using the two methods and randomly distributed to the physicians to ensure that the physicians and patients were double‐blinded.

#### 2.1.2. Group

##### 2.1.2.1. Digital Chairside Design for RPD Fabrication

An intraoral scanner (TRIOS2 Pod, 3‐Shape, Denmark) was connected to a computer and power supply; the scanning head was calibrated and preheated, and the patient underwent intraoral scanning to obtain dentition and soft tissue information.

**Figure 2:**
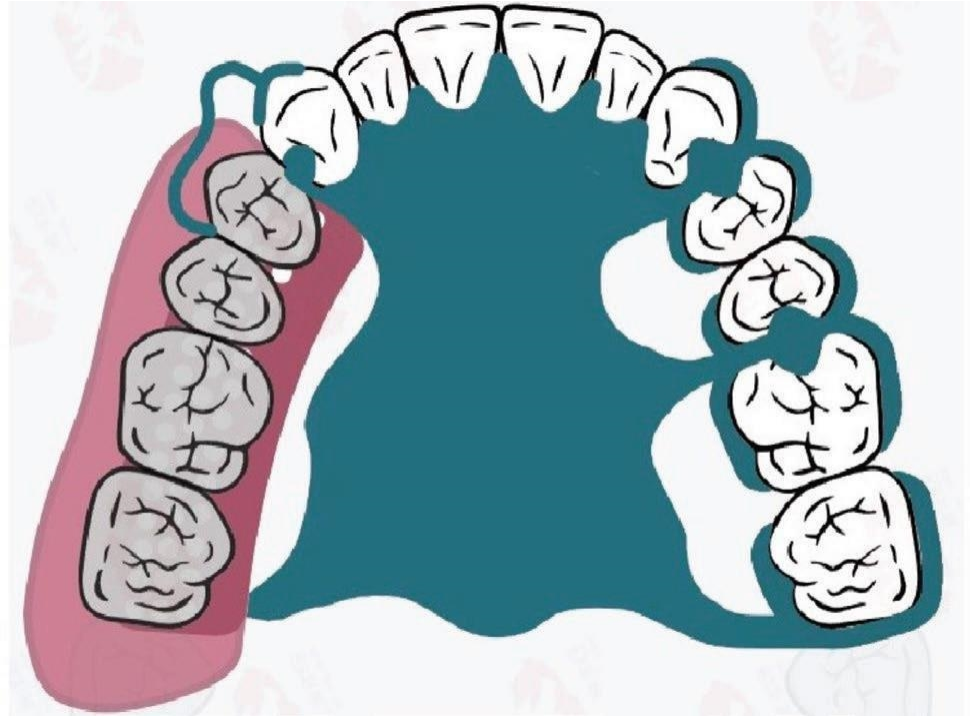
Preliminary design scheme.

**Figure 3:**
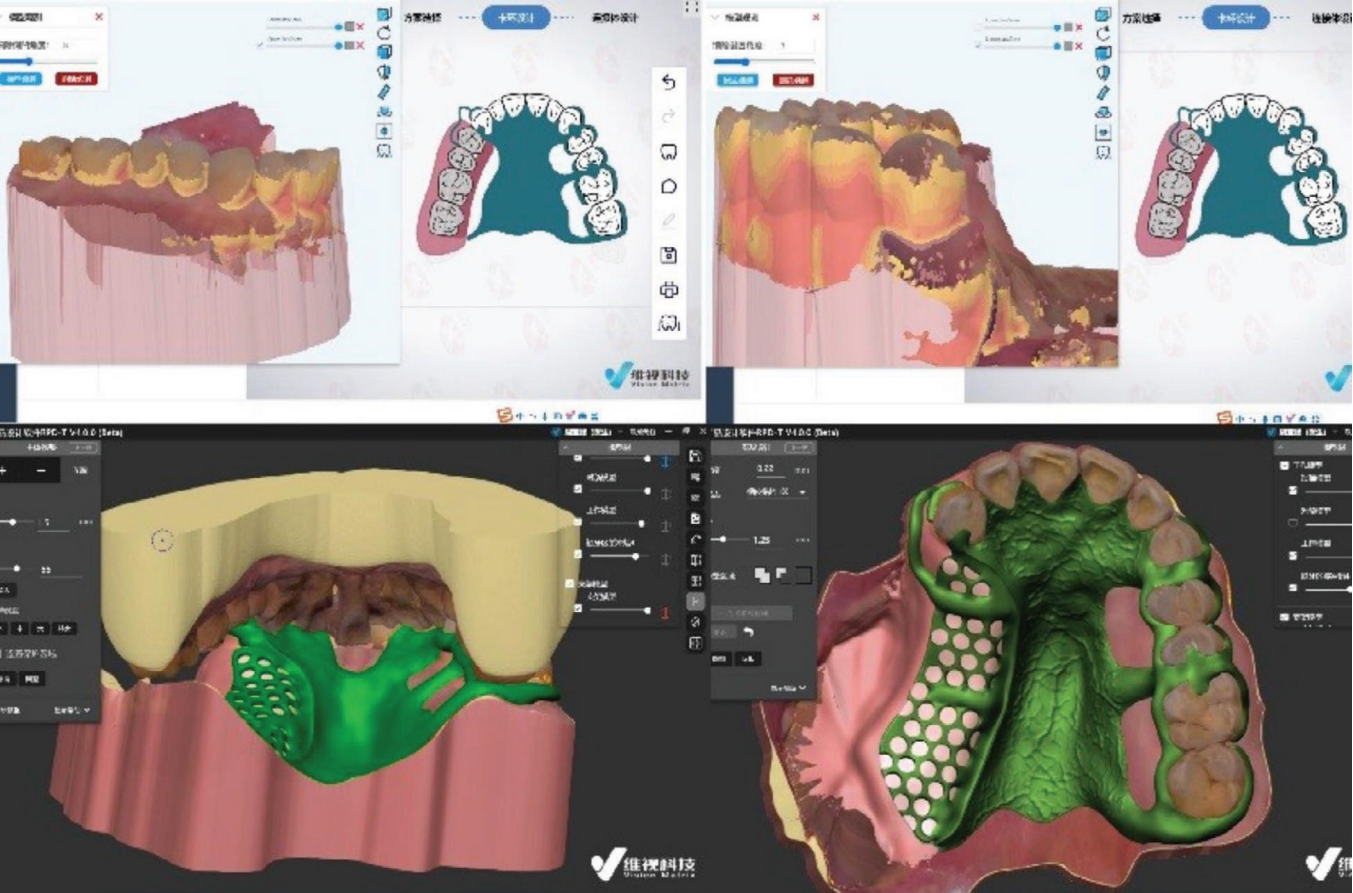
Completed model observations and jaw position transfer in 3D data management software.

A preliminary design scheme was generated by observing the model directly in the software and performing an undercut analysis (Figure 2). The physician adjusted and modified the design scheme according to the patient′s intraoral condition and completed intraoral tooth preparation according to the design scheme. The final impression and occlusal relationship data were obtained via a second oral scan (Figure 3).

The final impression data were sent to the technician for complete fabrication of the RPD framework using a 3D printing technique.

**Figure 4:**
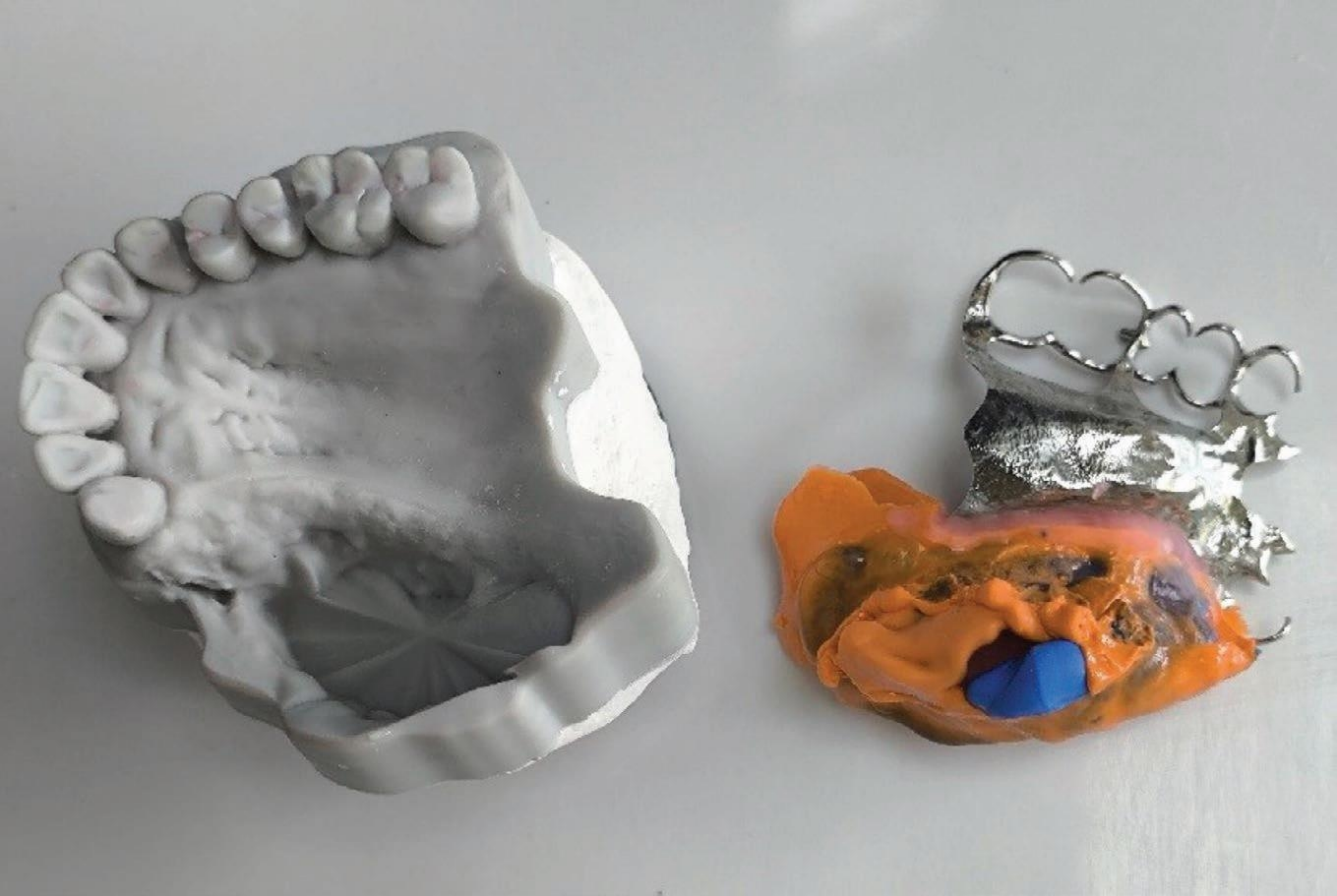
Fabrication of the RPD framework and preparation of the prosthesis plug model on a secondary basis.

The gutta‐filling glue and defect cavity prosthesis were applied on the basis of the fabricated framework (Figure 4).

##### 2.1.2.2. Traditional Process for RPD Fabrication

At the time of initial diagnosis, the initial impression was prepared, and the perfused model was sent to the technician for model observation and drawing the survey line. The design and tooth preparation were performed according to the observation line, followed by the final impression, which was sent to the technician after the perfused model to complete the fabrication of the cast RPD.

## 3. Evaluation of Clinical Quality of RPD

The patient′s chairside time at presentation was recorded, including the fabrication of the two dentures. The dentures were fitted by two physicians, and the denture quality was evaluated according to the following table (Table 1).

**Table 1:** Denture clinical quality score sheet.

Score items	Score
Seating	1—yes; 2—no
Occlusal rest	1—fit well; 2—fit relatively; 3—not fit
Clasp	1—fit well; 2—fit relatively; 3—not fit
Anterior margin of major connector	1—fit well; 2—fit relatively; 3—not fit
Posterior margin of major connector	1—fit well; 2—fit relatively; 3—not fit
Lateral margin of major connector	1—fit well; 2—fit relatively; 3—not fit
Retention	1—moderate resistance; 2—less resistance; 3—high resistance; 4—no resistance
Stability	1—no displacement (< 1 mm); 2—minor displacement (1–2 mm); 3—large displacement (> 2 mm); 4—very unstable (unable to use)
Occlusal adjustment	1—yes; 2—no

### 3.1. Results

**Figure 5:**
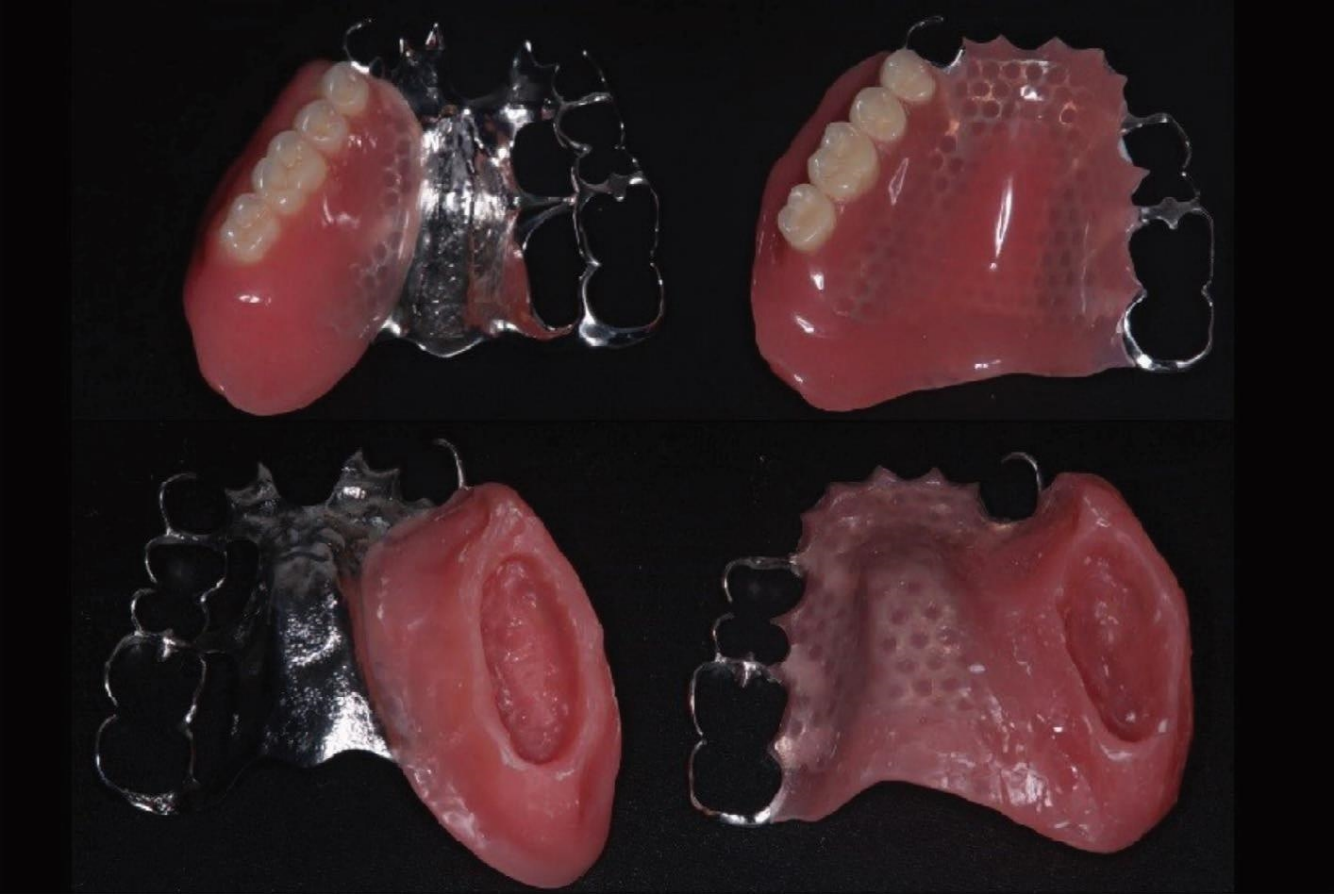
Final fabricated prosthesis (left: digital group; right: traditional group).

**Figure 6:**
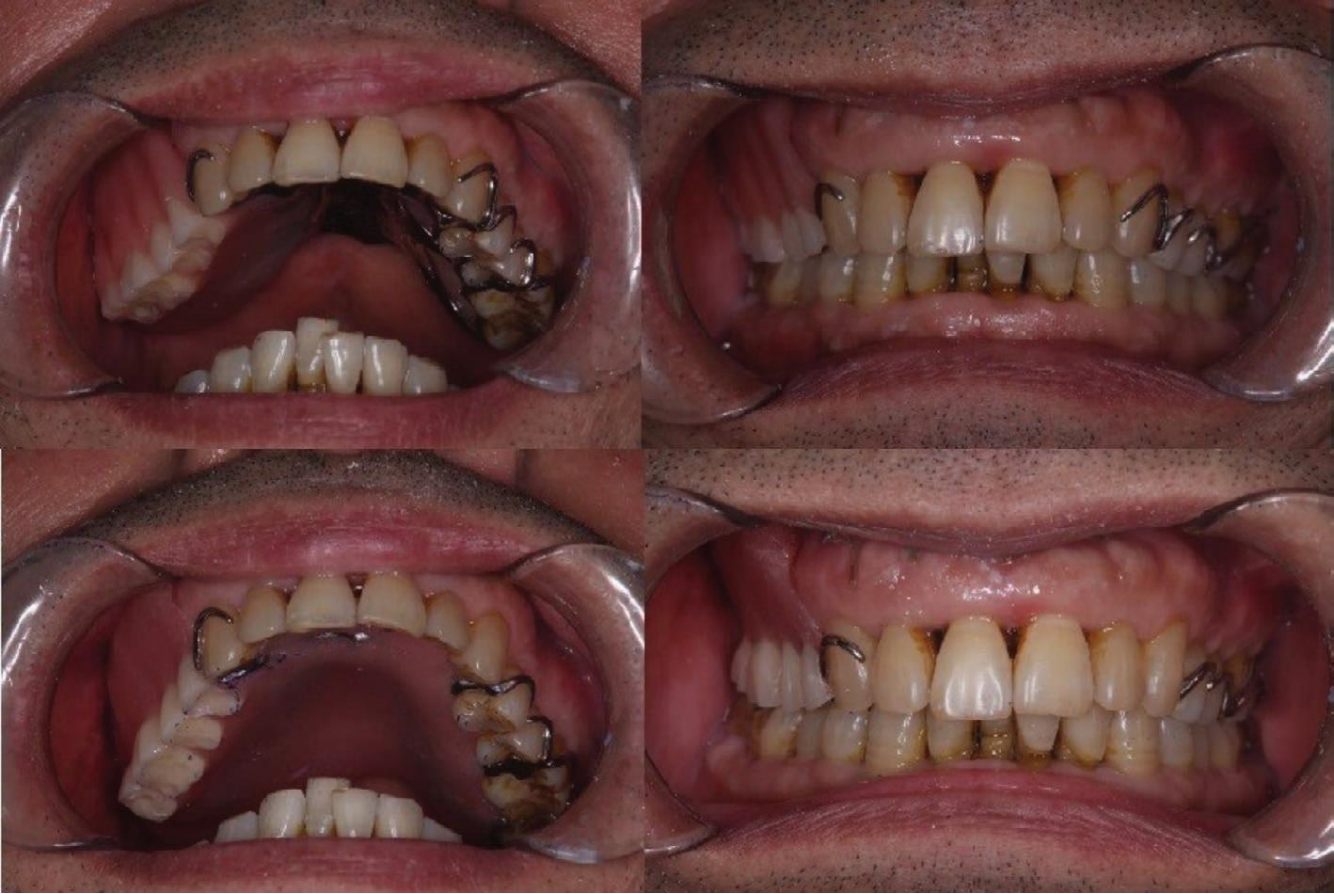
Intraoral trial of the prostheses (top: digital group; bottom: traditional group).

The prostheses fabricated using both methods achieved good restorative results (Figures 5 and 6).

In terms of the treatment process, the digital design and production process was obviously simpler than the traditional method, thus reducing the patient′s number of visits and chairside time (Table 2).

**Table 2:** Comparison of chairside times for the fabrication of dentures by different methods (A‐RPD framework made using the preliminary impression design; B‐RPD framework made using the digital chairside design).

Time (min)	Impression	Model surveying and guide‐line drawing	Framework fitting	Occlusal relationship record	Fitting and adjustment	Denture placement	Total
A	6.05	11.93	4.98	2.32	2.17	2.56	30.01
B	6.68^a^	/	/	/	/	4.15	10.83

^a^Impression of Group B includes the time for RPD design through the three‐dimensional management platform software.

In terms of the clinical quality of the fabricated RPDs, the frameworks fabricated using the digital design received positive evaluations from the physicians (Table 3).

**Table 3:** Physician evaluation of the two dentures fabricated using different methods (A‐RPD framework made using the preliminary impression design; B‐RPD framework made using the digital chairside design).

Score	Seating	Occlusal rest	Clasp	Anterior margin of major connector	Posterior margin of major connector	Lateral margin of major connector	Retention	Stability	Occlusal adjustment
A‐1	1	1	1	1	1	1	2	1	2
B‐1	1	1	1	1	1	1	1	1	1
A‐2	1	1	2	2	1	1	2	1	2
B‐2	1	1	1	1	1	1	1	1	1
A‐3	1	1	2	2	1	1	2	2	2
B‐3	1	1	1	1	1	2	2	1	1

## 4. Discussion

Rapid technological innovation has led to precise and rapid prosthodontic rehabilitation outcomes. Digital RPDs can be fabricated through the acquisition of intraoral scan data and the use of a three‐dimensional management platform to complete three‐dimensional observations. A three‐dimensional design can then be created, achieving data management, efficient medical technology use, and doctor–patient communication. A perfect digital repair solution compensates for the drawbacks of a traditional production process, including communication between doctors and technicians, data management, wire judgment, scheme drawing and other inconveniences. Thus, the use of a digital design improves the distribution of computer‐aided design (CAD), cutting/printing through computer‐aided manufacturing (CAM), and optimization of the production process while also improving product accuracy. Digital process repair not only improves production efficiency but also ensures the quality of the restoration and results in a good final repair effect [13]. In the clinical cases reported in this paper, RPDs fabricated using the digital chairside design and fabrication process were well evaluated in terms of marginal adaptation, retention, and stability. Moreover, the digital design resulted in optimization of the fabrication process, thereby decreasing the number of appointments and shortening the chairside time of patients.

Owing to technical limitations, tooth arrangement and gum filling must be completed manually; thus, full digitization of the RPD fabrication process cannot be realized. In addition, scanning of the mucosal support area increases the risk of soft tissue deformation, making it difficult to completely replace a pressure impression. We hope that the optimizations achieved by using a digital design can be expanded and that the full digitization of RPD production can be realized.

When clinicians are familiar with intraoral scanning equipment and digital observation software, the chairside time is greatly shortened. The results of the experiment indicate that the dentures observed and designed digitally can meet the requirements of clinical application. Digital technology has shown potential for simplifying treatment processes and enhancing patient comfort during treatment. Digital observation achieves model‐free RPDs, making model observation easier and intuitive while increasing the data storage time and increasing convenience.

The case reported in this article integrates three steps—digital impression taking, chair‐side observations, and RPD design—into an efficient communication process between doctors and technicians that can be completed within a single visit. This approach not only simplifies the treatment process but also enhances the efficiency and quality of RPD production. In the future, we will further refine our experiments and use more detailed data and statistical analyses to confirm the advantages of this process.

## Author Contributions

Su Wu and Ting Pan contributed equally to this work.

## Funding

This work was funded by the Key Specialty Construction Project of Shanghai Pudong New Area Health Commission (Grant No. PWZzb2022‐17).

## Consent

Written informed consent was obtained prior to prosthodontic treatment.

## Conflicts of Interest

The authors declare no conflicts of interest.

## Data Availability

The data that support the findings of this study are available from the corresponding authors upon reasonable request.

## References

[1] Douglass W. C. , Shih A. , and Ostry L. , Will There Be a Need for Complete Dentures in the United States in 2020?, Journal of Prosthetic Dentistry. (2002) 87, no. 1, 5–8, 10.1067/mpr.2002.121203, 11807476. 11807476

[2] Petropoulos V. C. and Rashedi B. , Removable Partial Denture Education in U.S. Dental Schools, Journal of Prosthodontics. (2006) 15, no. 1, 62–68, 10.1111/j.1532-849X.2006.00071.x, 16433654. 16433654

[3] Brawek P. K. , Wolfart S. , Endres L. , Kirsten A. , and Reich S. , The Clinical Accuracy of Single Crowns Exclusively Fabricated by Digital Workflow--The Comparison of Two Systems, Clinical Oral Investigations. (2013) 17, no. 9, 2119–2125, 10.1007/s00784-013-0923-5, 23371756. 23371756

[4] Syrek A. , Reich G. , Ranftl D. , Klein C. , Cerny B. , and Brodesser J. , Clinical Evaluation of All-Ceramic Crowns Fabricated From Intraoral Digital Impressions Based on the Principle of Active Wavefront Sampling, Journal of Dentistry. (2010) 38, no. 7, 553–559, 10.1016/j.jdent.2010.03.015, 20381576. 20381576

[5] Berrendero S. , Salido M. P. , Valverde A. , Ferreiroa A. , and Pradíes G. , Influence of Conventional and Digital Intraoral Impressions on the Fit of CAD/CAM-Fabricated All-Ceramic Crowns, Clinical Oral Investigations. (2016) 20, no. 9, 2403–2410, 10.1007/s00784-016-1714-6, 26800669. 26800669

[6] Gjelvold B. , Chrcanovic B. R. , Korduner E. K. , Collin-Bagewitz I. , and Kisch J. , Intraoral Digital Impression Technique Compared to Conventional Impression Technique. A Randomized Clinical Trial, Journal of Prosthodontics. (2016) 25, no. 4, 282–287, 10.1111/jopr.12410. 26618259

[7] Lin W. S. , Harris B. T. , and Morton D. , The Use of a Scannable Impression Coping and Digital Impression Technique to Fabricate a Customized Anatomic Abutment and Zirconia Restoration in the Esthetic Zone, Journal of Prosthetic Dentistry. (2013) 109, no. 3, 187–191, 10.1016/S0022-3913(13)60041-4, 23522368. 23522368

[8] Mansour M. , Sanchez E. , and Machado C. , The Use of Digital Impressions to Fabricate Tooth-Supported Partial Removable Dental Prostheses: A Clinical Report, Journal of Prosthodontics. (2016) 25, no. 6, 495–497, 10.1111/jopr.12346, 26371612. 26371612

[9] Hu F. , Pei Z. , and Wen Y. , Using Intraoral Scanning Technology for Three-Dimensional Printing of Kennedy Class I Removable Partial Denture Metal Framework: A Clinical Report, Journal of Prosthodontics. (2019) 28, no. 2, e473–e476, 10.1111/jopr.12712, 29143451. 29143451

[10] Zhong Y. J. , Li Z. Q. , Liu Y. Y. , Fan L. L. , and Zhang N. , Effects of Digital Impression on Clinical Adaptation of Removable Partial Dentures Restoring Dentition Defect of Kennedy Class II, Journal of Oral and Maxillofacial Prosthetics. (2022) 23, 111–114, 10.19748/j.cnki.kqhmxfx.2022.02.007.

[11] Su T. S. , Tang Y. , and Sun J. , Application of Intraoral Scanning and 3D Printing in the Manufacture of Removable Partial Dentures, Chinese Journal of Tissue Engineering Research. (2020) 24, 544–548, 10.3969/j.issn.2095-4344.2259.

[12] Huang J. , Mei Z. , Huang G. , Guo Y. , and Meng X. , Application of Digital Impression and Model in Removable Partial Dentures for Kennedy classIandIIdentition Defects, West China Journal of Stomatology. (2024) 42, no. 4, 481–485, 10.7518/hxkq.2024.2024103, 39049636. 39049636 PMC11338494

[13] Wang Y. J. and Wei B. , Current Status of the Application of Digital Impression Technology in Removable Partial Dentures, Tomatology. (2024) 44, 462–468, 10.13591/j.cnki.kqyx.2024.06.012.

